# Effects of six kinds of sperm staining methods on human sperm size and evaluation of their staining effects

**DOI:** 10.1002/jcla.24794

**Published:** 2022-11-28

**Authors:** Yuan‐Hua Xu, Jin‐Chun Lu, Shan‐Shan Tang

**Affiliations:** ^1^ Center for Reproductive Medicine Zhongda Hospital, Southeast University Nanjing China

**Keywords:** human sperm, morphological analysis, sperm size, staining method, standardization

## Abstract

**Background:**

Large‐ and small‐headed sperm are common morphological abnormalities. If different sperm staining methods affect sperm size, they will make a difference in the accuracy of sperm morphological analysis results. In this case, the normal reference values of sperm head parameters for different staining methods should be established.

**Methods:**

Six sperm staining methods, including Papanicolaou, Diff‐Quik, Shorr, Hematoxylin–eosin (HE), Wright, and Wright‐Giemsa staining, were used to stain the sperm smears of 25 semen samples, respectively. Sperm head parameter's length (L), width (W), area (A), perimeter, acrosomal area (Ac), and the derived values L/W and Ac/A of 2500 sperm (100 for each specimen) per staining method were measured by a computer‐aided sperm morphological analysis system.

**Results:**

The highest sperm head length and width were observed with the Wright‐Giemsa and Wright staining, followed by the Diff‐Quik. The lowest sperm head length and width were observed with the Papanicolaou staining, and the sperm head length and width of HE and Shorr staining were between those of Papanicolaou and Diff‐Quik staining. There was the same trend in changes in sperm head area and perimeter. Diff‐Quik and Shorr staining could clearly distinguish acrosome and nucleus, followed by HE staining, whereas the boundary between acrosome and nucleus was not evident in Papanicolaou, Wright, and Wright‐Giemsa staining.

**Conclusion:**

Different staining methods influence sperm size, and the normal reference values of sperm head parameters of each staining method should be established. Diff‐Quik and Shorr staining may be suitable methods for routine sperm morphological analysis.

## Graphical abstract

The highest sperm head length, width, area, and perimeter were observed with the Wright‐Giemsa and Wright staining, followed by the Diff‐Quik, Hematoxylin–eosin, and Shorr staining, and the lowest sperm head size was observed with the Papanicolaou staining. The staining effects of the six sperm staining methods were also different. Diff‐Quik and Shorr staining could clearly distinguish acrosome and nucleus, followed by HE staining, while the boundary between acrosome and nucleus was not evident in Papanicolaou, Wright, and Wright‐Giemsa staining.

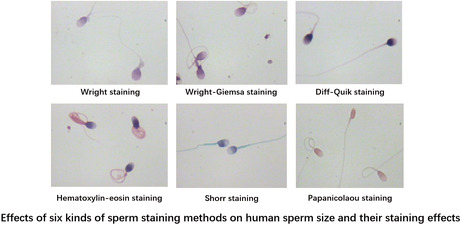

## 1 INTRODUCTION

The analysis of sperm morphology can be used to predict sperm fertilizing ability and spontaneous conception status,^1^ especially the overall analysis of sperm head, middle piece, and tail, along with the patient's living habits, occupation, and clinical manifestations, may contribute to the primary diagnosis of the patient's reproductive potential.^2^ It can also be employed to assess the reproductive toxicity of different physical and chemical factors, the effects of the treatment of semen samples, and the exploration of the possible etiology of teratozoospermia.^3^ Oocyte fertilization can be achieved by the technologies of intracytoplasmic sperm injection (ICSI), motile sperm organelle morphology examination (MSOME), and intracytoplasmic morphologically selected sperm injection (IMSI), which may somewhat weaken the clinical application of sperm morphology analysis. However, the standardized procedure, the practice of quality control, and accumulated experience in the analysis of sperm morphology can significantly improve the application value of sperm morphology analysis in clinical diagnosis and treatment.^4^

Sperm morphological analysis is one of the critical indices for evaluating sperm quality.^5^ Although there are reports on the morphological analysis of live sperm,^1^, ^6^, ^7^ routine sperm morphological analysis is still based on the evaluation of stained sperm. In addition to Papanicolaou staining, Diff‐Quik staining, and Shorr staining recommended by the World Health Organization (WHO),^8^, ^9^ Hematoxylin–eosin (HE) staining, Wright staining, and Wright‐Giemsa staining are also used for sperm morphological analysis in some laboratories.^10^

Large‐ and small‐headed sperm are common morphological abnormalities. It was reported that acute fever,^11^ ambient air pollution (especially an increase in fine particulates ≤2.5 μm),^12^ and meiotic abnormalities during spermatogenesis would lead to a high percentage of small‐headed sperm. A high percentage of large‐headed sperm was more likely to be associated with an incomplete partition of homologous chromosomes during meiosis I and of sister chromatids during meiosis II,^13^ and some gene mutations such as aurora kinase C (AURKC) gene.^14^, ^15^ Large‐headed spermatozoa were associated with a high rate of chromosomal abnormalities, polyploidy, diploidy, aneuploidy, and DNA fragmentation.^16^ Small‐ and large‐headed spermatozoa presented a high degree of noncondensed chromatin.^17^ During routine semen analysis, when small‐headed sperm were found in more than 70%, whether in vitro fertilization (IVF) or ICSI was performed, the fertilization rate in the cycles with small‐headed sperm was significantly lower than that with normal sperm heads.^18^ An increase in the proportion of large‐headed sperm was commonly associated with a low chance of pregnancy^13^ and even led to recurrent miscarriage.^19^ Therefore, sperm size should be accurately assessed. If different sperm staining methods affect sperm size, they will make a difference in the accuracy of sperm morphological analysis results.

WHO manual provided the normal sperm head length and width of Papanicolaou staining,^8^, ^9^ and some computer‐aided sperm morphological analysis (CASMA) systems also used these values to judge normal sperm. However, it is unclear whether different staining methods affect sperm size and which staining method is more suitable for routine sperm morphological analysis. If different sperm staining methods affect sperm size, the normal reference values of sperm head parameters for different staining methods should be established. Based on these, six sperm staining methods commonly used in China, including Papanicolaou, Diff‐Quik, Shorr, Hematoxylin–eosin (HE), Wright, and Wright‐Giemsa staining, were used to stain the sperm smears of 25 semen samples, respectively. Then, sperm head parameters length (L), width (W), area (A), perimeter, acrosomal area (Ac), and the derived values ratios of L to W (L/W) and Ac to A (Ac/A) of 2500 sperm (100 for each specimen) per staining method were measured by a CASMA system. The detection results and staining effects of different staining methods were compared. The detailed research process is as follows.

## 2 MATERIALS AND METHODS

### 2.1 Instruments and reagents

The CFT‐9202 computer‐aided sperm morphological analysis (CASMA) system was purchased from Jiangsu Rich Life Science Instrument Co., Ltd. Papanicolaou staining solution, Diff‐Quik staining solution, Shorr staining solution, Hematoxylin–eosin (HE) staining solution, Wright staining solution, and Wright‐Giemsa staining solution were purchased from Zhuhai Beisuo Biological Technology Co., Ltd.

### 2.2 Sample sources

Semen samples were obtained from men of infertile couples by masturbation. Twenty‐five semen samples with good liquefaction and normal sperm concentration were selected. The surplus part of these samples after routine semen analysis was used for this study.

### 2.3 Preparation of sperm smears

Two milliliters of fresh liquefied semen were washed twice with normal saline by centrifugation for 5 min at 600*g*. Then, sperm pellets were resuspended with an appropriate amount of normal saline according to the initial sperm concentration of semen samples to ensure that the sperm concentration in these suspensions was between 20 × 10^6^/ml and 50 × 10^6^/ml. Eight smears were prepared for each sperm suspension sample, of which six were used for six kinds of different staining, and the other two were spare for preventing problems in staining or operation. When preparing a sperm smear, a drop of sperm suspension was first put on a clean slide by a dropper. Then the excess sperm suspension was removed by sucking from the drop's center to the surrounding. During the process of sucking back, the end of the dropper should be flat, the dropper should be vertical to the slide, and the excess suspension should be slowly sucked back. The prepared sperm smears were dried in the air for the subsequent sperm staining.

### 2.4 Sperm staining

The dried sperm smears were stained according to the instructions for six kinds of staining solutions, respectively. The operation details of each staining method are as follows.

#### 2.4.1 Papanicolaou staining

The dried sperm smears were fixed in 95% alcohol for 15 min. Then, the smears were immersed in 75% ethanol, 50% ethanol, and water for 30 s, respectively. Next, the smears were stained with hematoxylin staining solution (Harris) for 5 min and washed with water for 1 min. Subsequently, the smears were differentiated in 1% hydrochloric acid alcohol solution for several seconds and returned to blue in running water for 10 min. Then, the smears were immersed in 50% ethanol, 75% ethanol, and 95% ethanol for 30 s, 30 s, and 2 min, respectively. Next, the smears were stained with orange G staining solution for 1 min and immersed in 95% ethanol twice for 15 s each time. Afterward, the smears were stained with EA36 staining solution (bright green and eosin) for 5 min. Last, the smears were immersed in 95% ethanol twice for 15 s and anhydrous ethanol twice for 45 s each time.

#### 2.4.2 Diff‐Quik staining

First, the dried sperm smears were immersed in solution A containing methanol and eosin for 30 s. Next, the smears were immersed in phosphate buffer solution to wash off solution A. The smears were immersed in solution B containing methylene blue for 30 s and then washed with water.

#### 2.4.3 Shorr staining

First, the dried sperm smears were fixed in the solution containing methanol and triarylmethane for 1 min and washed lightly with running water. Next, the smears were stained with hematoxylin solution (Harris) for 3 min and washed lightly with running water. Then, the smears were immersed in acidic ethanol for 10 s and washed with running water for 2 min. After the smears were immersed in 50% ethanol for 1 min, they were stained with Shorr staining solution for 1 min and washed with running water. Last, the smears were immersed in 50% ethanol, 95% ethanol, and 95% anhydrous ethanol for 1 min, respectively.

#### 2.4.4 HE staining

First, the dried sperm smears were stained with hematoxylin staining solution (Harris) for 5 min and washed with running water for 1 min. Next, the smears were differentiated in 1% hydrochloric acid alcohol solution for several seconds and returned to blue in running water for 10 min. Then, the smears were stained with eosin solution for 1 min and washed lightly with running water. Last, the smears were immersed in 95% ethanol twice for 10 s and anhydrous ethanol twice for 1 min each time.

#### 2.4.5 Wright staining

About 1 ml of Wright staining solution was added to the dried sperm smears, and the smears were stained for 1 min. Then, two times of phosphate buffer solution (pH 6.8) were added to the smears, and the two solutions were mixed well with an ear‐washing bulb. After the smears were stained for 10 min, they were washed with running water.

#### 2.4.6 Wright‐Giemsa staining

About 1 ml of Wright‐Giemsa staining solution was added to the dried sperm smears, and the smears were stained for 1 min. Then, two times of phosphate buffer solution (pH 6.8) were added to the smears, and the two solutions were mixed well with an ear‐washing bulb. After the smears were stained for 10 min, they were washed with running water.

After the stained smears were dried in the air, the measurement of sperm head parameters and the evaluation of sperm staining effects were performed under the microscope with an oil immersion objective.

### 2.5 Measurement of sperm head parameters

Sperm head parameters were measured by a CASMA system. Before measuring sperm head parameters, the CASMA system was calibrated according to the requirements of the analysis software to verify the accuracy of the scale of the system. After that, the staining effects of different sperm staining methods were evaluated under a microscope with an oil immersion objective. Sperm head length (L), width (W), area (A), perimeter (P), acrosomal area (Ac), and the derived values L/W and Ac/A of 2500 sperm (100 sperm for each specimen) per staining method were determined after being magnified by the CASMA. To ensure the accuracy of the measurement results, each parameter was manually rechecked or corrected.

### 2.6 Statistical analysis

The data were expressed as mean ± SD and analyzed with the SPSS 22.0 statistical software (SPSS Inc). The comparisons of sperm head length (L), width (W), area (A), perimeter (P), L/W and Ac/A of different staining methods were performed by paired t test, and *p* ≤ 0.05 was considered to be statistically significant.

## 3 RESULTS

### 3.1 Comparisons of sperm head length (L), width (W), area (A), perimeter (P), L/W, and Ac/A of six kinds of staining methods

The sperm head length and width of Wright‐Giemsa staining were significantly higher than those of the other five staining methods (*p* < 0.001, Table 1) through the comparisons of sperm head length (L), width (W), area (A), perimeter (P), L/W and Ac/A of 2500 sperm from 25 samples (100 sperm from each sample) for different staining methods. The sperm head length and width of Wright staining were close to that of Diff‐Quik staining, and both of them were significantly lower than that of Wright‐Giemsa staining (*p* < 0.001) but higher than those of Papanicolaou, Shorr, and HE staining (*p* < 0.001). The sperm head length and width of HE staining were close to that of Shorr staining, and both of them were significantly lower than those of Wright, Wright‐Giemsa, and Diff‐Quik staining (*p* < 0.001) but higher than that of Papanicolaou staining (*p* < 0.001). The lowest sperm head length and width were observed with Papanicolaou staining, significantly lower than the other five staining methods (*p* < 0.001). In general, there was no apparent difference in sperm head L/W between different staining methods. Although statistical analysis showed that the sperm head L/W of Wright‐Giemsa staining was the lowest, which was significantly lower than those of Wright (*p* = 0.002), Diff‐Quik (*p* < 0.001), and Shorr staining (*p* = 0.01), and that the sperm head L/W of Papanicolaou staining was significantly lower than that of Diff‐Quik staining (*p* = 0.046), there was no significant difference in sperm head L/W among other staining methods (Table 1). The sperm head area and perimeter of Wright‐Giemsa staining were also the highest, significantly higher than those of the other five staining methods (*p* < 0.001, Table 2). There was no significant difference in sperm head area and perimeter between Wright and Diff‐Quik staining, and both of them were only lower than that of Wright‐Giemsa staining. The sperm head area and perimeter of HE, Shorr, and Papanicolaou staining decreased in turn, which was significantly lower than those of Wright‐Giemsa, Wright, and Diff‐Quik staining (*p* < 0.001), and there were significant differences between them (*p* < 0.01). There were significant differences in sperm Ac/A between six kinds of staining methods (*p* < 0.001, Table 2).

**TABLE 1:** Comparison of sperm length (L), width (W), and L/W for six kinds of sperm staining methods (mean ± SD, *n* = 2500).

Staining methods	Length (μm)	Width (μm)	L/W
Wright‐Giemsa	4.73 ± 0.65	2.98 ± 0.44	1.61 ± 0.31
Wright	4.57 ± 0.62*	2.84 ± 0.43*	1.64 ± 0.42**
Diff‐Quik	4.57 ± 0.64*	2.82 ± 0.40*	1.64 ± 0.32*
Hematoxylin–eosin	4.42 ± 0.61* ^,^ ^†^ ^,^ ^‡^	2.75 ± 0.37* ^,^ ^†^ ^,^ ^‡^	1.63 ± 0.31
Shorr	4.39 ± 0.60* ^,^ ^†^ ^,^ ^‡^	2.73 ± 0.39* ^,^ ^†^ ^,^ ^‡^	1.63 ± 0.32^††^
Papanicolaou	4.20 ± 0.57* ^,^ ^†^ ^,^ ^‡^ ^,^ ^§^ ^,^ ^¶^	2.63 ± 0.40* ^,^ ^†^ ^,^ ^‡^ ^,^ ^§^ ^,^ ^¶^	1.62 ± 0.35^‡‡^

* *p* < 0.001 versus Wright‐Giemsa staining.

^†^ *p* < 0.001 versus Wright staining.

^‡^ *p* < 0.001 versus Diff‐Quik staining.

^§^ *p* < 0.001 versus Hematoxylin–eosin staining.

^¶^ *p* < 0.001 versus Shorr staining.

** *p* = 0.002 versus Wright‐Giemsa staining.

^††^ *p* = 0.01 versus Wright‐Giemsa staining.

^‡‡^ *p* = 0.046 versus Diff‐Quik staining.

**TABLE 2:** Comparison of sperm area (A), perimeter (P), and acrosomal area (Ac)/A for six kinds of sperm staining methods (mean ± SD, *n* = 2500).

Staining methods	Area (μm^2)^	Perimeter (μm)	Acrosomal area/area (%)
Wright‐Giemsa	11.10 ± 2.49	12.82 ± 1.49	26.40 ± 10.24
Wright	10.22 ± 2.27*	12.34 ± 1.42*	28.46 ± 10.15*
Diff‐Quik	10.16 ± 2.09*	12.34 ± 1.40*	23.77 ± 8.61* ^,^ †
Hematoxylin–eosin	9.57 ± 1.98* ^,^ ^†^ ^,^ ^‡^	11.94 ± 1.34* ^,^ ^†^ ^,^ ^‡^	24.92 ± 9.58* ^,^ ^†^ ^,^ ^‡^
Shorr	9.41 ± 1.94* ^,^ ^†^ ^,^ ^‡^ ^,^ ^¶^	11.85 ± 1.32* ^,^ ^†^ ^,^ ^‡^ ^,^ ^††^	27.37 ± 9.57* ^,^ ^†^ ^,^ ^‡^ ^,^ ^§^
Papanicolaou	8.75 ± 2.04* ^,^ ^†^ ^,^ ^‡^ ^,^ ^§^ ^,^ **	11.38 ± 1.33* ^,^ ^†^ ^,^ ^‡^ ^,^ ^§^ ^,^ **	29.46 ± 7.49* ^,^ ^†^ ^,^ ^‡^ ^,^ ^§^ ^,^ **

* *p* < 0.001 versus Wright‐Giemsa staining.

^†^ *p* < 0.001 versus Wright staining.

^‡^ *p* < 0.001 versus Diff‐Quik staining.

^¶^ *p* = 0.003 versus Hematoxylin–eosin staining.

^§^ *p* < 0.001 versus Hematoxylin–eosin staining.

** *p* < 0.001 versus Shorr staining.

^††^ *p* = 0.008 versus Hematoxylin–eosin staining.

### 3.2 Evaluation of staining effects for six kinds of sperm staining methods

**FIGURE 1:**
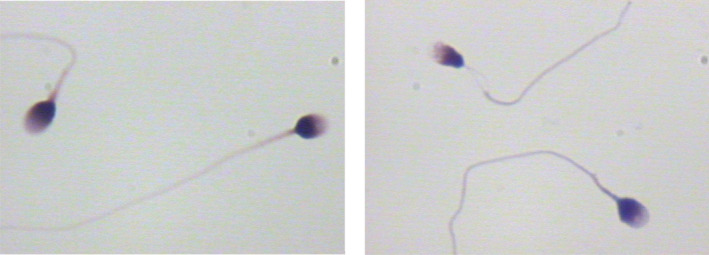
Sperm images for Diff‐Quik staining.

**FIGURE 2:**
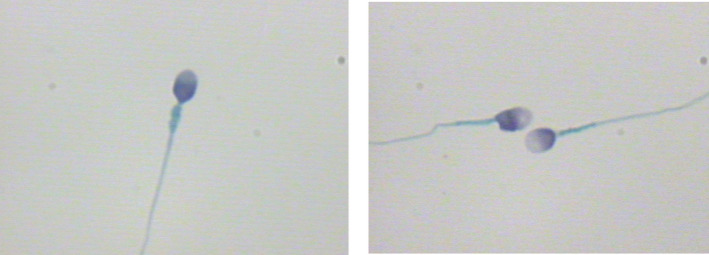
Sperm images for Shorr staining.

**FIGURE 3:**
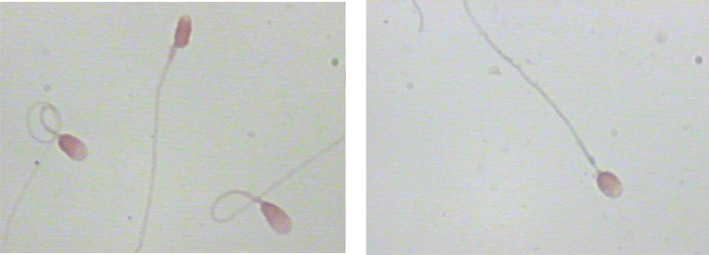
Sperm images for Papanicolaou staining.

**FIGURE 4:**
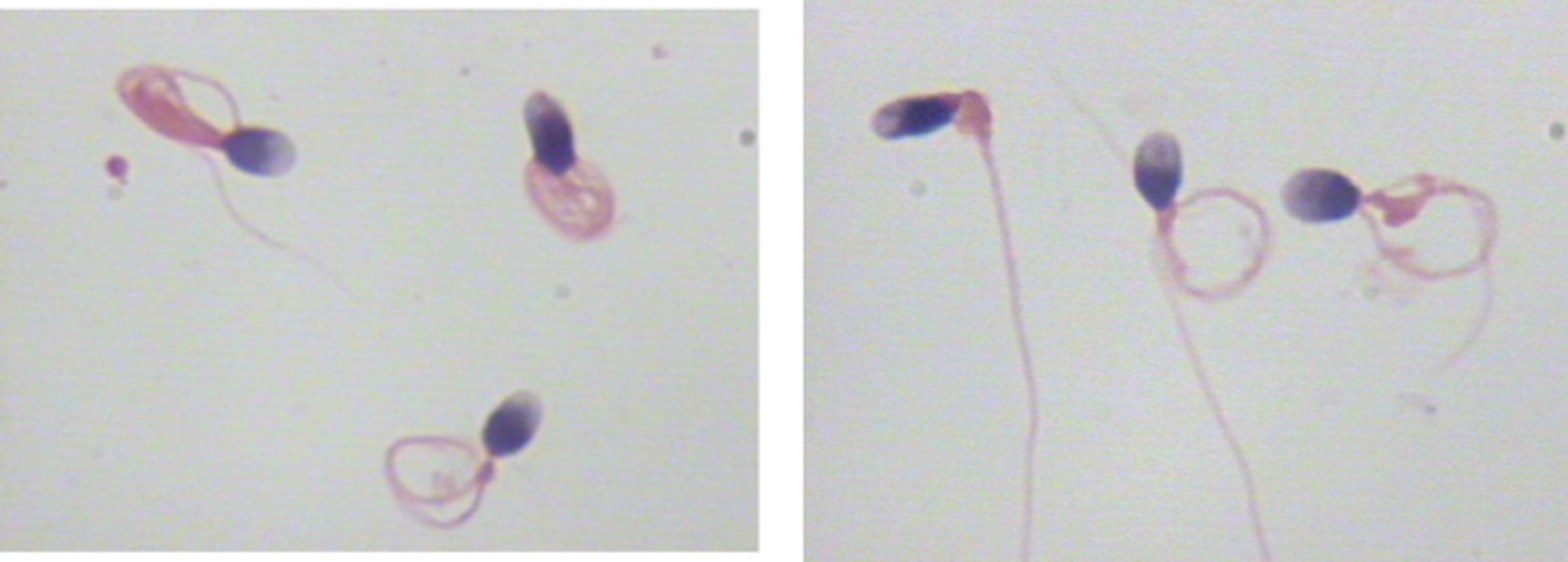
Sperm images for Hematoxylin–eosin staining.

**FIGURE 5:**
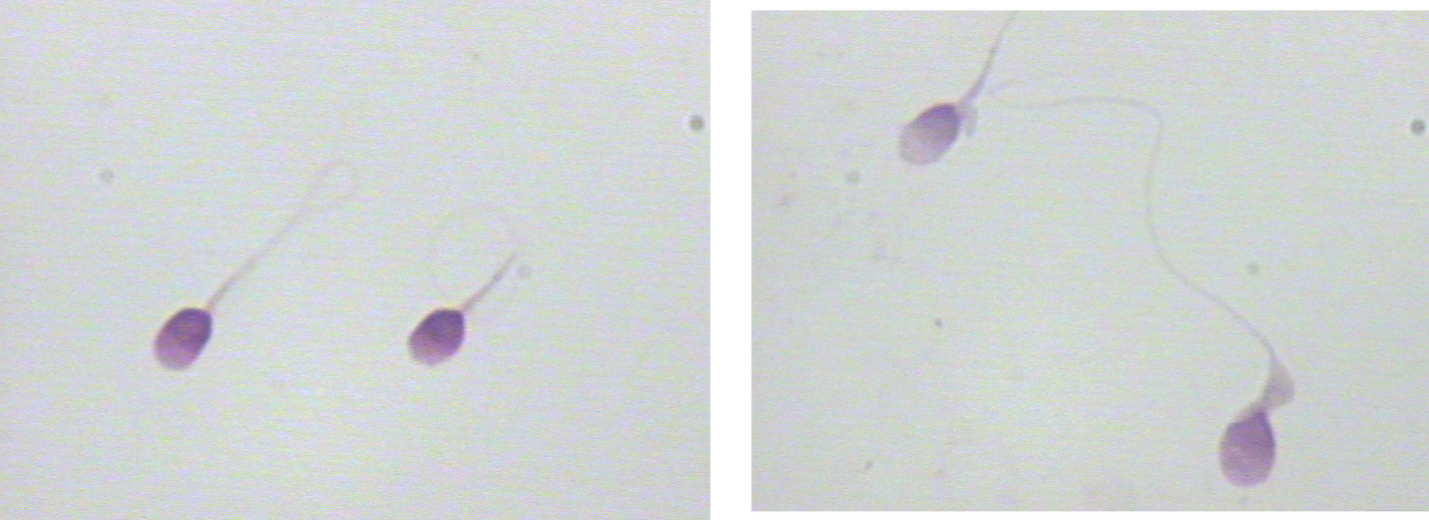
Sperm images for Wright staining.

**FIGURE 6:**
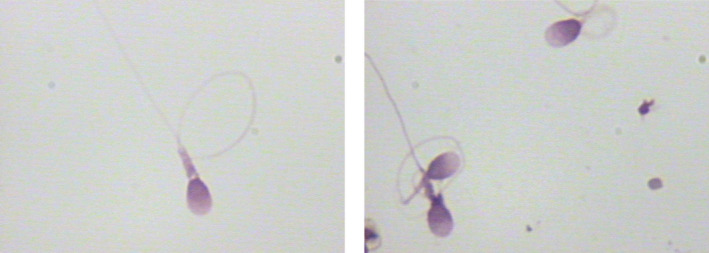
Sperm images for Wright‐Giemsa staining.

When comparing the sperm head parameters of six kinds of sperm staining methods, we also evaluated and analyzed the staining effects of different sperm staining methods carefully. The staining effects of six kinds of sperm staining methods were different (Figures 1, 2, 3, 4, 5, 6). Diff‐Quik and Shorr staining could clearly distinguish acrosome and nucleus, followed by HE staining, while the boundary between acrosome and nucleus was not evident in Papanicolaou, Wright, and Wright‐Giemsa staining. Six kinds of sperm staining methods could clearly show the neck and tail of sperm, especially Shorr staining.

## 4 DISCUSSION

Sperm morphology can be analyzed by a microscope or a CASMA system, and the CASMA system is increasingly widely used. The CASMA system can significantly increase the number of sperm analyzed and objectively classify sperm by measuring sperm parameters. Especially for the analysis of abnormal and normal sperm head size, technicians cannot distinguish slight differences and must rely on a CASMA system.^20^ Specified sperm morphological abnormalities, such as sperm head with vacuoles, may affect the outcome of assisted reproductive technology (ART),^21^ while large or small‐headed sperm may be related to abnormal meiosis of spermatogenic cells.^13^ Previous studies showed that protamine‐deficient sperm could significantly change the size and shape of sperm heads.^22^ So as to determine whether the size of the sperm head is normal or not, a CASMA system must have specific reference values of normal human sperm head length, width, area, perimeter, Ac/A, and L/W. However, only sperm head dimensions of Papanicolaou staining were reported.^8^, ^9^, ^20^ At present, multiple sperm staining methods are used in clinical laboratories. Are the sperm head parameters of Papanicolaou staining also suitable for other sperm staining methods? After an extensive literature search, there are few studies on sperm size after staining with different staining methods, and there is no relevant report on the comparison of sperm size after staining with different staining methods. Based on these, we compared the effects of six sperm staining methods on sperm size.

It was reported that different buffer systems and pH of different staining methods had different effects on cell morphology, integrity, and contents,^23^, ^24^ which would lead to a change in cell size. During the process of staining, the hypotonic or hypertonic extracellular environment could change cell size.^25^, ^26^ Especially, hypotonic solutions could lead to membrane leakage,^27^ which further promoted water and dye molecules to enter cells and cells to be colored. Different chemicals and dyes in staining solutions were able to change the size and shape of the nucleus after binding with the nucleus,^28^, ^29^ and the changes of intracellular ions could also lead to cell swelling or contraction.^30^, ^31^ In addition, the production of artifacts for different sperm staining methods,^1^ the quality of an optical system, and the settings of a CASMA system may also affect the analysis of sperm head size. Therefore, different staining methods may have different effects on sperm head size.

The results of this study showed that different staining methods had different effects on sperm head size. The larger sperm head length and width of Wright‐Giemsa and Wright staining might be related to the hypotonic environment of Wright‐Giemsa and Wright staining solutions resulting in the swelling of the sperm head. The methanol solution for dissolving Wright dye is hypotonic, and the phosphate buffer solution is isotonic. They will form a hypotonic solution after mixing. The lowest sperm head length and width of Papanicolaou staining may be related to dehydration in staining.^1^, ^29^ In fact, the length and width of sperm heads stained with the staining methods containing dehydration processes, such as Papanicolaou, Shorr, and HE staining, were significantly lower than that without dehydration processes such as Wright, Wright‐Giemsa and Diff‐Quik staining. Our team once measured the size of sperm heads without any staining. The results showed that the size of unstained sperm heads was close to that of Diff‐Quik staining (unpublished data), indicating that a hypotonic environment or dehydration in the process of staining might affect the size of sperm heads. In addition, the difference in sperm head size caused by different staining methods may also relate to the effects of different dyes on the internal composition or structure of sperm and sperm membranes, and the detailed mechanism needs further investigation.

The changing trend of sperm head area and perimeter of six kinds of staining methods was similar to that of sperm head length and width because the difference in sperm head length and width would inevitably lead to the change of sperm head area and perimeter. Bellastella et al.^20^ once measured the length, width, area, and perimeter of 7942 sperm heads stained by Papanicolaou staining solution using a CASMA system, and the results were slightly higher than the sperm head size measured in this study, which might be related to the different composition of sperm staining solution or the difference of research population.

The slight difference in sperm head L/W between the six kinds of sperm staining methods indicated that the effects of different staining methods on sperm were holistic. While the sperm head length was increased by a sperm staining method, the sperm head width was also increased correspondingly. Moreover, the difference in sperm head L/W between different staining methods may also be related to the morphological difference of different sperm. In addition, there were significant differences in sperm head Ac/A between six kinds of staining methods, and the order of sperm head Ac/A was as follows: Papanicolaou staining > Wright staining > Shorr staining > Wright‐Giemsa staining > HE staining > Diff‐Quik staining. It should be related to the ability of different staining methods to distinguish acrosome and nucleus. In addition, the analysis of sperm head Ac/A has certain subjectivity because when the boundary between the nucleus and acrosome is unclear, it is necessary to correct the boundary between the nucleus and acrosome manually. A sperm is a three‐dimensional cell, but what we observe is only the plane surface of the sperm. This plane surface is related to the angles of different observers, inevitably leading to the position difference between the nucleus and acrosome. In other words, when the same sperm is observed from different sides, its Ac/A will be different. Coupled with the influence of different staining methods, it is not difficult to understand the significant difference in sperm Ac/A between different staining methods. Therefore, sperm head Ac/A may be unsuitable for evaluating the effect of different staining methods on sperm morphology.

Because the sperm head and acrosome are the primary markers reflecting sperm morphology, the staining method which can accurately distinguish sperm acrosome and nucleus is relatively suitable for sperm morphological analysis. Our study showed that Diff‐Quik and Shorr staining could clearly distinguish acrosome and nucleus, followed by HE staining. However, the boundary between acrosome and nucleus was not evident in Papanicolaou, Wright, and Wright‐Giemsa staining. Moreover, most CASMA systems captured sperm based on the grayscale of sperm in the analysis of sperm morphology at present. Although the WHO manual recommended Papanicolaou staining for sperm morphological analysis,^8^, ^9^ our research showed that the sperm stained by Papanicolaou staining was challenging to be captured by a CASMA system, which was obviously inferior to the other five sperm staining methods. In addition, Papanicolaou staining was time‐consuming. Similarly, HE staining was also relatively time‐consuming. Therefore, based on the effects of different staining methods on sperm head size, staining effect, and the simplicity of operation, Diff‐Quik, and Shorr staining may be suitable methods for routine sperm morphological analysis. What needs to be further determined is the normal reference range of the length, width, area, perimeter, L/W, and Ac/A of sperm head measured by the two staining methods. Because there are few completely normal sperm in each semen sample, it is difficult to determine these parameters by large sample size. However, these parameters are essential for a CASMA system to judge whether sperm is normal or not. In addition, it is crucial to establish the natural dimensions of the unstained sperm head before determining the optimal staining method, and its reference values.^32^ In the future, it is possible to determine these parameters by staining the normal sperm screened by zona pellucida.

## 5 CONCLUSION

Our study demonstrated that different staining methods have different effects on the analysis of sperm head parameters, indicating that the normal reference values of sperm head parameters of each staining method should be established. Based on the effects of six sperm staining methods on sperm head size, staining effect, and the simplicity of operation, we believe that Diff‐Quik staining and Shorr staining are more suitable for routine sperm morphological analysis, especially for a CASMA system, which can be used as standard methods for sperm morphological analysis.

## AUTHOR CONTRIBUTIONS

Lu JC designed the study, analyzed the data, and wrote the paper; Yuan‐Hua Xu performed the research and wrote the paper; Shan‐Shan Tang performed the research. All authors read and approved the final manuscript.

## CONFLICT OF INTEREST

All authors declare no competing interests.

## Data Availability

The data that support the findings of this study are available from the corresponding author upon reasonable request.
